# Ampulla of Vater carcinoma: advancement in the relationships between histological subtypes, molecular features, and clinical outcomes

**DOI:** 10.3389/fonc.2023.1135324

**Published:** 2023-05-18

**Authors:** Hao Liang, Yu Zhu, Ya-kun Wu

**Affiliations:** Department of Hepatobiliary Surgery, Suining Central Hospital, Suining, China

**Keywords:** ampulla of Vater carcinoma, molecular features, clinical outcomes, relationships, histological subtypes

## Abstract

The incidence of ampulla of Vater carcinoma, a type of periampullary cancer, has been increasing at an annual percentage rate of 0.9%. However, patients with ampulla of Vater carcinoma have quite different prognoses due to the heterogeneities of the tissue origin of this carcinoma. In addition to TNM staging, histological subtypes and molecular features of ampulla of Vater carcinoma are the key factors for predicting the clinical outcomes of patients. Fortunately, with the development of testing technology, information on the histological subtypes and molecular features of ampulla of Vater carcinoma is increasingly being analyzed in-depth. Patients with the pancreaticobiliary subtype have shorter survival times. In immunohistochemical examination, high cutoff values of positive MUC1 staining can be used to accurately predict the outcome of patients. Mutant *KRAS*, *TP53*, negative *SMAD4* expression, and microsatellite stability are related to poor prognosis, while the clinical value of *BRCA1/BRCA2* mutations is limited for prognosis. Testing the histological subtypes and molecular characteristics of ampulla of Vater carcinoma not only is the key to prognosis analysis but also provides extra information for targeted treatment to improve the clinical outcomes of patients.

## 1 Introduction

Ampulla of Vater carcinoma (AVC) arises from the complex periampullary region, including the common bile duct, duodenum, and Wirsung duct, and thus, has a mixed tissue origin (1). The incidence rate of AVC is 0.49 per 10^5^ individuals (2, 3). Importantly, the incidence rate of AVC has been increasing (3).

Currently, the standard treatment for AVC is controversial. Pancreaticoduodenectomy (PD) and subsequent chemotherapy, similar to the therapeutic plan for pancreatic adenocarcinoma, have been utilized in many AVC cases (4). However, in contrast to the single tissue origin of pancreatic adenocarcinoma, treatments for AVC may need to depend on the tissue origin of AVC because of the complex anatomical structures of the ampulla of Vater. The substantial heterogeneity of AVC, including complex histological subtypes and molecular features, may be an important reason for different prognoses in different patients.

With the development of testing technology such as immunohistochemical examinations, exome sequencing, and ultradeep sequencing, the analysis of data regarding histological subtypes and molecular features of AVC has been performed in recent years. This promoted the clarification of the pathological mechanism of AVC and the classification of groups of patients with a high risk of death. Importantly, this information could guide further therapy to some degree and influence the prognosis of AVC patients.

In this article, a comprehensive review of recent studies regarding AVC was conducted, especially focusing on the relationship between histological subtypes, molecular features, and clinical outcomes.

## 2 Ampulla of Vater and AVC

### 2.1 Ampulla of Vater

The ampulla of Vater, called the ampulla complex, constitutes the dilated junction of the biliary, pancreatic, and digestive tracts, and it lies in the wall of the descending duodenum (5). In the ampulla of Vater, the terminal common bile duct and Wirsung duct are surrounded by the sphincteric system of Oddi, terminating at the major duodenal papilla, which are covered by the duodenal mucosa. The junction of the terminal common bile duct and Wirsung duct in the major duodenal papilla has three variations: 1) separate duodenal openings for the two canals, 2) a double-barreled opening at the apex of the papilla, and 3) an opening in the common duct (5).

The tissues from the ampulla complex, namely, intestinal, pancreatic ductal, and biliary epithelial tissues, have morphological heterogeneities (6). Therefore, when AVC is suspected, the tissue origin of AVC should be assessed.

### 2.2 AVC

AVC is a type of rare malignancy, and the current official data fail to show its epidemiological characteristics (7, 8). A large retrospective study from the United States reported that the age-adjusted incidence rate of AVC was 7.4 per 1,000,000 person-years between 1999 and 2013 (9). From the Biliary Tract Cancers Pooling Project of 19 studies (10), the incidence rates of AVC in women reached 12 per 1,000,000 person-years between 1980 and 2017.

However, the incidence rate of AVC for all racial groups has been significantly increasing at an annual percentage rate of 0.9% between 1973 and 2005 (*P* < 0.05) (3). Sometimes, the diagnosis of AVC and cancer of the head of the pancreas may be confusing. From another angle, AVC is a frequent indication for PD, accounting for approximately 21%–30% of diagnoses in patients receiving this procedure (11, 12). Between January 1995 and December 2014, nearly 21.6% (110/510) of patients who received PD therapy were finally diagnosed with AVC, second only to cancer of the head of the pancreas (11). In patients with periampullary cancers receiving PD therapy (12), the proportion of patients diagnosed with AVC was as high as 29.5% (61/207) during 1998–2009. In addition, the National Comprehensive Cancer Network (NCCN) published its first version of clinical practice guidelines for AVC on 9 March 2022.

Therefore, paying more attention to this special group of patients in surgical practice is cost-effective, as the proportion of AVC cases in patients undergoing PD therapy increases.

### 2.3 Different clinical outcomes of AVC and pancreatic cancer

Although the standard curative treatment for both pancreatic cancer and AVC is PD, patients with AVC have a much better overall survival (OS) than those with pancreatic cancer. The median survival of patients with resected pancreatic cancer was only 14.3 months, and the 5-year OS rate was 20.5% in a multicenter, phase III trial (13). However, the median OS of patients with resected AVC was 64 months and the 5-year OS rates were as high as 53% in a retrospective multicenter cohort study of 887 patients (14), and these results were consistent with those of other studies (51%–58%) (15–17).

The high OS may be related to certain features (18) of AVC, namely, significantly smaller tumor sizes and lower prevalence of lymph node metastases (all *P* < 0.001) as well as the PD therapy itself. Nevertheless, the strong influence of different histological subtypes (19) and some special genetic mutations (20) on the clinical outcomes of AVC patients should be noted, and the NCCN guidelines mention the relationship of histological subtypes, molecular features, and clinical outcomes.

## 3 Histological subtypes of AVC associated with clinical outcomes

### 3.1 Four histological subtypes of AVC

Importantly, the histological classification of solid tumors can be used to predict the clinical outcome of patients (12). According to the anatomical features of the ampulla of Vater, the tissue origin of AVC can be divided into three histological subtypes: the pancreaticobiliary, intestinal, and mixed subtypes (**Table 1**). Additionally, an unknown histological subtype of AVC has been reported. Interestingly, a study reported that the mixed subtype of AVC could be classified according to the predominant type of tissue, but there were 108 (33.9%) patients with unknown histological subtypes (21). In total, related reports about unknown histological subtypes of AVC are rare. Therefore, the pancreaticobiliary and intestinal subtypes are mainly discussed in this article, and the mixed subtype of AVC is mentioned.

**Table 1:** Key definitions concerning histologic classification.

	Definitions and functions
Pancreaticobiliary subtype	A type of histomorphology of ampulla of Vater carcinoma resembles the histomorphology features of pancreatic cancer
Intestinal subtype	A type of histomorphology of ampulla of Vater carcinoma resembles the histomorphology features of colon adenocarcinoma
Mix subtype	A complex type of histomorphology of ampulla of Vater carcinoma with pancreatic and colon adenocarcinoma tissues. This type is often categorized based on the predominant subtype
Unknown subtype	A rare reported special type of histomorphology of ampulla of Vater carcinoma with unknown original tissues
CDX2	Caudal type homeobox 2, a major regulator of lots of intestine-specific genes involved in cell growth and differentiation
MUC1	Mucins 1, a membrane-bound protein that could promote tumor progression, regulate transcription, and determine cell fate in the genotoxic stress response
MUC2	Mucins 2, coating the epithelia of the intestines and other mucus membrane-containing organs, providing a protective, lubricating barrier against particles and infectious agents at the mucosa
MUC5AC	Mucins 5AC may be an extracellular matrix structural constituent, which protects the mucosa from damage by binding to harmful microorganisms and particles
CK20	Cytokeratin 20, playing an important role in maintaining keratin filament organization in intestinal epithelia
CK7	Cytokeratin 7, expressed in the simple epithelia in the gland ducts, could block the interferon-dependent interphase and stimulate DNA synthesis in cells
CK17	Cytokeratin 17, expressed in the nail bed, hair follicle, and sebaceous glands
TNM staging	It refers to the extent of the cancer, including the size of the tumor (T), the invaded lymph nodes (N), and the invaded parts of the body (M)

### 3.2 Proportion of the two histological subtypes

Previous reports have shown that the proportions of the pancreaticobiliary and intestinal subtypes in AVC are variable. An international multicenter retrospective cohort study showed that there was a great proportion of pancreaticobiliary subtype AVC patients (293/547, 53.6%), while the proportion of intestinal subtype AVC patients was 38.6% (211/547), and only 43 AVC patients had the mixed subtype (14). Another retrospective study of 99 patients (18) also reported that there were slightly more patients with the pancreaticobiliary subtype than the intestinal subtype (49.5% *vs*. 44.4%). Another retrospective study (21) revealed that the number of patients with the pancreaticobiliary subtype was approximately equal to that with the intestinal subtype (*n* = 105 *vs*. *n* = 106). This study classified the mixed subtype of AVC into the intestinal subtype or pancreaticobiliary subtype. Another study (22) with a total of 45 patients between March 2015 and December 2019 revealed that the number of AVC patients with the pancreaticobiliary subtype was lower than that with the intestinal subtype (46.8% *vs*. 53.2%). The limited samples in the study may have contributed to the above results.

The number of AVC patients with the pancreaticobiliary subtype accounts for a large number of patients, and the 5-year OS of AVC patients with the pancreaticobiliary subtype is only 47% (14). Thus, its negative influence on the outcome of the whole group of AVC patients should be noted.

### 3.3 Pancreaticobiliary subtype related to poor prognosis

The pancreaticobiliary subtype is significantly associated with poor prognosis (**Table 2**), which has been validated in several different studies (12, 17, 18, 21–30), including meta-analyses. A large-sample research (21) showed that the median disease-free survival (DFS) time of patients with the intestinal subtype was 58.9 months. However, for those with the pancreaticobiliary subtype, DFS was only 25.3 months (*P* = 0.0123). The reasons may be that patients with the pancreaticobiliary subtype tend to present with a more advanced T stage and more frequently with regional lymph node involvement and perineural invasion than those with the intestinal subtype (15, 21, 23, 26, 28). Furthermore, a retrospective study of 106 patients (25) analyzed the influence of postoperative complications of PD therapy and the positive node ratio on prognosis from 1996 to 2015, and compared with the intestinal subtype, the pancreaticobiliary subtype was associated with poor prognosis (HR = 2.11, 95% CI = 1.04–4.27, *P* = 0.04). Furthermore, a previous study of 232 patients (31) indicated that AVC patients with the pancreaticobiliary subtype had a much better prognosis than those with pancreatic cancer (41 *vs*. 15.6 months, *P* < 0.001).

**Table 2:** Information of those articles concerning the histological subtypes of ampulla of Vater carcinoma and clinical outcomes.

	Types	Time of study	Sample sizes	Histological subtypes, PB/IT	Pancreaticobiliary subtype related to poor prognosis, HR; (*P*)
Westgaard A et al., 2013	Retrospective	1998 to 2009	61	23/36	2.68; (0.009)
Schueneman A et al., 2015	Retrospective	1992 to 2007	163	93/70	2.26; (0.0009)
Robert PE et al., 2014	Retrospective	1990 to 2011	319	105/106	2.0; (0.0013)
Doepker MP et al., 2016	Retrospective	1996 to 2015	106	30/50	2.80; (0.01)
Leo JM et al., 2016	Retrospective	1987 to 2013	99	49/44	NA; (0.0156)
Zhou Y et al., 2017	Meta-analysis	2000 to 2016	2,234	899/1,021	1.93; (0.004)
Palmeri M et al., 2020	Retrospective	2009 to 2016	21	10/3	8.39; (0.0152)
Nappo G et al., 2021	Retrospective	2010 to 2018	97	54/34	NA; (0.004)
Xia T et al., 2021	Retrospective	2015 to 2019	47	18/27	6.633; (0.006)
Fernandez-Placencia RM et al., 2022	Retrospective	2010 to 2020	83	19/57	2.7; (0.025)

PB, pancreaticobiliary subtype of ampulla of Vater carcinoma; IT, intestinal subtype of ampulla of Vater carcinoma; HR, hazard ratio; NA, not applicable.

### 3.4 Role of immunohistochemical examination in prognosis

Currently, there is no clear standard to discriminate the different histological subtypes of AVC *via* only morphological features or combined with immunohistochemical examination. Exploring the auxiliary use of immunohistochemical examination to define the histological subtypes of AVC and predict the clinical outcome is ongoing (19).

A group of patients with a pancreaticobiliary subtype (CDX2 negative and MUC1 positive in immunohistochemical examination) and lymph node metastases had a median survival of 11.9 months, and there were no 5-year survivors in this study of 208 patients from three independent cohorts (19). Considering that MUC1 was the key marker for diagnosing the pancreaticobiliary subtype in previous reports, a study of 163 patients from 1992 until 2007 further showed that a higher cutoff value (≥10%) for defining positive MUC1 staining may have better discrimination power for patients with a significantly worse prognosis (17).

In addition, immunohistochemical examination (**Table 1**) may play a role in the management and treatment of AVC patients with mixed subtypes. A retrospective study of 99 patients showed that AVC patients with mixed subtypes (negative MUC1, MUC2, CDX2, and CK20) had a similar prognosis to those with the pancreaticobiliary subtype (18). Another study with 97 patients (23) validated the result and described that patients with mixed subtypes had high 1-, 3-, and 5-year OS rates (87.5%, 33.3%, and not reached), similar to those with the pancreaticobiliary subtype (93.7%, 66.9%, and 53.6%, *P* = 0.4).

Furthermore, some other new key biomarkers for immunohistochemical examination have been assessed. High expression of CK7 and negative expression of CK20 in patients with the pancreaticobiliary subtype were revealed by tissue microarray chip analysis (27, 28). Additionally, the positive rate of CK17 expression in patients with the pancreaticobiliary subtype was very high (20/22, 90.9%) (28). More interestingly, MUC5AC positivity in AVC patients may have a direct and strong correlation with the clinical outcome. Regardless of histological subtype (32), setting the cutoff of MUC5AC positivity at either >25% or 1% (any positivity was regarded as an expression) and testing it on all patients yielded significant survival differences between the MUC5AC-positive *versus* MUC5AC-negative patients (*P* = 0.0007 and *P* = 0.02).

While the sample sizes in those studies were limited, new biomarkers from immunohistochemical examination should be developed and applied for predicting clinical outcomes in the future.

### 3.5 Other opinions on histological subtypes and outcomes

There seem to be other opinions on the effects of the pancreaticobiliary subtype on clinical outcome. No difference in median OS was found between intestinal subtype patients and pancreaticobiliary subtype patients (33). This result may be attributed to the limited sample sizes (45 cases), and this research was mainly focused on the somatic and germline genetic alterations of AVC patients. A retrospective cohort study showed that N stage, not the pathological subtype, was a stronger predictor of OS (14), which may be mainly due to the higher ratio of patients with the pancreaticobiliary subtype receiving adjuvant chemotherapy. The other reason may be that pancreaticobiliary subtype patients tended to present with a more advanced T stage and more frequently with N stage and perineural invasion than intestinal subtype patients (15).

TNM staging is a critical predictive factor for the clinical outcomes of AVC patients. A study reported the development of a prognostic nomogram for AVC patients based on the Surveillance, Epidemiology, and End Results database, which supported the strong influence of TNM staging on the outcome of AVC patients (26). However, it did not include data on chemotherapy, major comorbidities, and other important factors that could affect prognosis. Additionally, it failed to analyze the prognosis of patients with different histological subtypes. Moreover, a study that built a prognostic score for AVC patients receiving PD therapy (34, 35) based on TNM staging did not show close relationships between the pancreaticobiliary subtype and prognosis (1, 36). Furthermore, in the eighth edition guidelines, the T category has been reconsidered based on the strong prognostic impact of TNM staging, including the depth of duodenal and pancreatic invasion (37).

It is difficult to infer the causal relationship between the histological subtypes and other high-risk factors for death, such as TNM staging. Nevertheless, it is obvious that TNM staging may have a strong independent impact on the prognosis of AVC. However, while it might be weak, the influence of different histological subtypes on clinical outcomes should be noted, as it exists and might be independent.

### 3.6 Limitations of histological classification for AVC

Based on the different histological subtypes, patients with a high risk of death could be screened. However, it is difficult to select the optimal therapy and ultimately improve the outcome. Additionally, there are some histological subtypes without a clear definition, such as the mixed and unknown subtypes. One retrospective study indicated that the mixed subtype of AVC might have a distinct tumor nature and a significantly poor clinical outcome (38).

## 4 Molecular classification associated with clinical outcomes

Identifying the molecular characteristics of AVC could be very interesting and impactful. The molecular classification of AVC is another important supplement for histological classification. Molecular classification could overcome the shortcomings of histological classification, as it can be used to predict the prognosis of patients and provide doctors with useful guidance for performing proper chemotherapy, targeted treatment, and immunotherapy (39). Rapid advances in sequencing technologies, including panel sequencing, exome sequencing, and ultradeep sequencing, have permitted in-depth analysis for the characterization of the molecular profile of AVC. Recently, the molecular features of other types of cancer, such as gastric cancer, have been estimated. Therefore, the NCCN guidelines for AVC also suggest testing for inherited mutations in all confirmed AVC patients, including *TP53*, *MLH1*, *MSH2*, *MSH6*, *BRCA1*, *BRCA2*, and *CDKN2A* (**Table 3**).

**Table 3:** Key definitions concerning molecular classification.

	Definitions and functions
*TP53*	Tumor protein 53 gene, as a cellular stress sensor, activated by the conditions including DNA damage, improper mitogen stimulation, and oncogene activation
*MLH1*	MutL Homolog 1, a tumor-suppressor gene involved in DNA mismatch repair
*MSH2/MSH6*	MutS Homolog 2/6 gene, binding to DNA mismatches and initiating DNA repair
*BRCA1/BRCA2*	Breast cancer susceptibility gene 1/2, playing a role in transcription, DNA repair of double-stranded breaks, and recombination
*CDKN2A*	Cyclin-dependent kinase inhibitor 2A, a tumor-suppressor gene, triggering cell cycle arrest and senescence, and apoptosis regulator genes
*KRAS*	The Kirsten rat sarcoma viral oncogene, managing the signal transduction from membrane receptors to intracellular molecules, controlling multiple cellular functions such as proliferation and apoptosis
*SMAD4*	Drosophila mothers against decapentaplegic gene, inducing cell cycle arrest and apoptosis at the early stages of tumor formation
*BAX*	Expressing the BCL2-associated X protein which increases the opening of the mitochondrial voltage-dependent anion channel, leading to the release of cytochrome c and apoptosis
*MET*	Mesenchymal to epithelial transition factor gene, regulating many physiological processes including proliferation, scattering, morphogenesis, and survival
Raf–MEK–ERK	A key signaling pathway in the regulation of cell proliferation, differentiation and survival, and intense research on the pathways for the development of pharmacologic inhibitors for cancer therapy
PI3K–AKT–mTOR	A hyperactivated signaling pathway for regulating the cellular processes including survival, proliferation, growth, metabolism, angiogenesis, and metastasis

### 4.1 Different types of genetic mutations in AVC

The genetic mutations associated with AVC are very complex (40). *KRAS* mutations are oncogenic mutations. *TP53* and *BRCA2* mutations lead to alterations in tumor suppressors, which are closely related to the tumorigenesis of AVC. Gene mutations related to the mismatch repair of DNA (*MLH1*, *MSH2*, *MSH6*) are also tested in AVC patients, and they could cause microsatellite instability (MSI). Mutations in a cell cycle-related gene, *CDKN2A*, have been identified in AVC patients. *SMAD4* mutations could lead to alterations in cell signaling proteins, which also correlated with the clinical outcomes of AVC patients. The alterations in these molecules are summarized and discussed in this review (**Table 4**). There are many additional changes in the molecular characteristics of AVC patients, including *MDM2*, *ERBB2*, *ELF3*, and *PIK3CA* (33, 41–43), but the reported clinical values of these gene changes in AVC patients may be limited.

**Table 4:** The molecular features with clinical values in ampulla of Vater carcinoma.

	Frequency	Common location of the mutation	Common histological subtypes	Prognosis	Treatments
*KRAS* mutation	45%	Codon 12 genotypes	Pancreaticobiliary subtype	OS, RFS, and DFS	Sotorasib
*TP53* mutation	36%	NA	Intestinal subtype	OS and RFS	PC14586
*BRCA1/BRCA2* mutation	12.5%	NA	Pancreaticobiliary subtype and intestinal subtype	OS^a^	Rucaparib
*SMAD4* mutation	6%	NA	Pancreaticobiliary subtype and intestinal subtype	OS^b^	NA
Microsatellite instability	8.3%	*MLH1*, *MSH2*, *MSH6*	Intestinal subtype	OS^a^	Pembrolizumab

OS, overall survival; RFS, recurrence-free survival; DFS, disease-free survival; NA, not applicable.

a Better prognosis.

b Without statistical significance.

### 4.2 KRAS mutation in AVC patients

The Kirsten rat sarcoma viral oncogene (*KRAS*) is a key gene that manages signal transduction from membrane receptors to intracellular molecules, activating the canonical Raf–MEK–ERK, PI3K–AKT–mTOR (**Table 2**), RalGDS–RalA/B, or TIAM1–RAC1 signaling pathways. The *KRAS* gene has the ability to control multiple cellular functions, such as proliferation, apoptosis, motility, and survival (44).

*KRAS* is one of the most frequently mutated genes in AVC patients (40). The mutation frequency of *KRAS* was as high as 33% in a study of 146 patients between 1982 and 2008 (45). A meta-analysis study (46) revealed that 45% (175/388) of AVC patients had *KRAS* mutations, which had a significant correlation with worse recurrence-free survival (HR = 2.74, *P* = 0.0008). A small sample study reported that *KRAS* was mutated in 35% of patients (47) and emerged as an independent prognostic factor regardless of the histological subtype (HR = 3.85, *P* = 0.0018). A large sample study showed that the median OS for AVC patients with mutant *KRAS* was 22.3 *versus* 47.7 months in patients carrying the wild-type *KRAS* gene (20).

Although there were overlapping genomic alterations between the two histological subtypes, a trend has been observed (42) that more frequent *KRAS* mutations are found in AVC patients with the pancreaticobiliary subtype (61%) than in AVC patients with the intestinal subtype (29%). When histological subtypes were analyzed together (48), concurrent *KRAS* gene positivity and *MET* copy number gain were proven to be independent prognostic factors for DFS in AVC patients with the pancreaticobiliary subtype (HR = 4.926, *P* = 0.047). This may contribute to the findings that *KRAS* mutation is significantly associated with patients presenting with a large tumor size (38).

Furthermore, there are some specific mutational genotypes of *KRAS*, especially codon 12 mutation—*KRAS* genotype G12C. The median survival time of patients with the *KRAS* genotype G12C mutation was 62 months, which was significantly poorer than that of patients without the *KRAS* mutation (155 months) in a retrospective study of 146 patients (45). The high mutation rate of *KRAS* genotype G12C could become a possible therapeutic target. Sotorasib (49), an inhibitor of *KRAS* genotype G12C, has just been approved by the Food and Drug Administration (FDA) to treat non-small cell lung cancer. Currently, other inhibitors for *KRAS* genotype G12C mutations, such as MRTX849, are being developed (50). These drugs may be beneficial for AVC patients with *KRAS* genotype G12C mutations in the future.

### 4.3 TP53 mutation in AVC patients

Tumor protein 53 gene (*TP53*) is a cellular stress sensor that is activated by conditions including DNA damage, improper mitogen stimulation, and oncogene activation (51). The downstream target genes transactivated by *TP53* are numerous, including cyclin-dependent kinase inhibitor 1A (*CDKN1A*, encoding p21), which triggers cell cycle arrest and senescence, and apoptosis regulator genes (*BAX* and *PUMA*), which trigger apoptosis (52).

*TP53* (encoding p53) is the other frequently mutated gene in patients with AVC. In all, 36%–72% of patients have been shown to have high levels of *TP53* gene expression (35, 47, 53–57). Another study reported that in 82.4% (56/69) of AVC patients, abnormal p53 immunolabeling was detected (38), including nuclear accumulation of immunolabeled p53 (41.2%) and lack of p53 immunolabeling (41.2%).

*TP53* mutation has been shown to be related to the clinical outcome of AVC patients. A previous study of 80 patients (47) showed that *TP53* mutation is a negative predictor of survival in AVC patients (HR = 3.85, *P* = 0.0006), regardless of histological subtype. In a retrospective study of 53 patients (58), the clinical prognosis of AVC patients with *TP53* overexpression was worse than that of the remaining patients (*P* = 0.006). Furthermore, a retrospective study, which consisted of 92 patients, reported that *TP53*-dependent transcription of cyclin-dependent kinase 1 was associated with an increased risk of recurrence-free survival (HR = 3.97, *P* = 0.02) and worse OS (HR = 11.16, *P* = 0.005) in patients with AVC after resection therapy (53).

Regarding the features of *TP53* mutations in different histological subtypes of AVC, the difference in the proportion of *TP53* mutations in the intestinal subtype and pancreaticobiliary subtype patients failed to reach statistical significance (55). A recent retrospective study (57) seemed to reveal that *TP53* mutation was more frequent in AVC patients with the intestinal subtype (27/46, 58.6%) than in those with the pancreaticobiliary subtype (13/37, 35.1%, *P* = 0.042). This may be attributed to the limited sample sizes and the proportion of different histological subtypes in those AVC patients. The frequency of *TP53* mutations in patients with different histological subtypes of AVC may need to be investigated in the future.

Currently, there are two chemical compounds, PC14586, a Y220C-selective structural corrector of mutant *TP53*, and BI 907828, an MDM2-p53 antagonist, in phase I clinical trials (59). Thus, a substantial amount of research must be conducted before these compounds can be applied in clinical practice.

### 4.4 BRCA1/BRCA2 mutation in AVC patients

Breast cancer susceptibility gene 1/2 (*BRCA1/BRCA2*) mutations predispose patients to select cancers, especially breast and ovarian cancer (40). However, a few studies have found *BRCA2* mutations in AVC patients (33, 60, 61).

The frequency of *BRCA2* mutations in AVC patients is low, but it might indicate the risk of cancer in female relatives of AVC patients. A small sample retrospective study showed that the frequency of *BRCA2* mutations was 12.5% (2/16) in AVC patients, and the relatives of two patients had a history of cancer (61). For the prognosis, 3 of 44 patients with available germline data had pathogenic alterations in *BRCA2*, but the OS of the 3 patients were clearly different—84, 18, and 2 months (33). Although it is an indispensable event for some cancers, *BRCA2* mutations appear to be biologically neutral in a proportion of other cancers (62), including AVC. Therefore, meaningful pathogenic germline variants of *BRCA2* should be identified further. In the different histological subtypes, the frequency of *BRCA2* mutations in the pancreaticobiliary subtype could be similar to that in the intestinal subtype (6/13, 46.2% *vs*. 11/22, 50.0%, *P* = 0.3) (60).

Additionally, testing the *BRCA2* mutation could guide doctors to use rucaparib (a type of poly ADP-ribose polymerase inhibitor, PARPi) for the treatment of patients with advanced ovarian cancer and metastatic castration-resistant prostate cancer (63). Therefore, AVC patients with loss of heterozygosity or mutations in *BRCA2* may benefit from the use of PARPi (33), and the clinical outcomes of those patients could be improved to some degree.

### 4.5 SMAD4 mutation in AVC patients

The *SMAD4* gene plays a pivotal role in the switch of TGF-β function by inducing cell cycle arrest and apoptosis at the early stages of tumor formation (64). The frequency of *SMAD4* mutation is quite variable, ranging from 6% to 75% (38, 43, 57, 65, 66). Furthermore, a retrospective study reported that two patients (10%) with mutant *SMAD4* were identified among 20 patients with *KRAS* mutation (43).

The clinical outcome of patients with *SMAD4* mutations might be similar to that of patients without mutations (40, 66). The mortality rate (66) was higher for AVC patients with negative (absent, trace, or focal) *SMAD4* expression (62% *vs*. 31%) than for patients with positive *SMAD4* expression, although the difference may not be statistically significant (*P* = 0.0865). The reason might contribute to the limited sample size—63 cases. However, an earlier study of 140 patients indicated that the *SMAD4* mutation was not related to the 5-year survival rate (67). However, there were no more detailed data in that article. The influence of *SMAD4* mutation on prognosis should be validated in a larger sample cohort study.

Regarding the histological subtypes, a previous retrospective study reported that the frequency of *SMAD4* mutations in AVC patients with the intestinal subtype (28.2%) was higher than that in patients with the pancreaticobiliary subtype (16.2%), but the *P*-value was 0.293 (57). The difference in the frequency was not significant (38, 67). In addition, there are currently no available drugs to target *SMAD4* mutations.

### 4.6 Microsatellite instability and AVC

MSI, a molecular phenotype of tumors, is the gain or loss of nucleotides in the short repeating motifs of DNA elements (microsatellite tracts) (68). It was highlighted that if the patient had tumors with MSI phenotypes, analysis of PD-1/PD-L1 expression or tumor mutational burden is unnecessary for receiving immunotherapy—immune checkpoint inhibitor treatments (69). The damaged mismatch repair system (MMR deficiency) for DNA, including the alteration in the *MLH1*, *MSH2*, or *MSH6* gene, could cause MSI. However, MMR deficiency often has a Lynch-suggestive profile, and routine testing for Lynch syndrome is warranted (29). MSI may be a dependable marker for prognosis.

The overall frequency of MSI phenotypes in AVC patients is unclear (70), but it might be lower than 8.3% (69). Four of 49 (8.2%) tested AVC patients showed MSI phenotypes in next-generation sequencing analysis and showed loss of *MLH1* (65). Two of 53 consecutive (6%) patients showed MMR deficiency (70). Despite the low frequency of MSI phenotypes (3%), patients with MSI phenotypes had a better survival than patients with non-MSI periampullary cancers (*P* < 0.0021). Additionally, the six AVC patients with MSI phenotypes survived from 2 to 8 years after diagnosis (41), which was higher than the AVC patients with microsatellite stability phenotypes (*P* = 0.04). AVC patients with MSI phenotypes who underwent resection therapy had no evidence of recurrent disease at the cutoff time in a study of 59 patients (65). The AVC patients (29) with MMR deficiency accounted for up to 18% (23/128). Regarding the relationship between histological subtypes and MSI, a study reported that both AVC patients with MSI had the intestinal subtype of AC (65).

Although current articles concerning AVC patients with MSI are limited and the data are discrepant, the value of MSI for immunotherapy is clear. Testing MSI, MMR deficiency, tumor mutational burden, and even the number of tumor-infiltrating lymphocytes could inform the choice of proper therapy for improving the clinical outcome of AVC patients.

### 4.7 Others

CD340 (*HER2*) amplification is not a rare event (14/106, 13%) in AVC patients, and it can be reliably identified using routine immunohistochemistry (42). Patients with CD340 amplification may be candidates for targeted therapy (trastuzumab). Other mutant genes have also been reported, although their clinical values are limited. Mutant *CDKN2A* in AVC patients has been reported (71). Several other molecular alterations in the PI3K, Wnt signaling, and TGF-β pathways and the cell cycle have also been reported in AVC patients (40). Activation of the AKT and MAPK pathways is increased in patients with the pancreaticobiliary subtype with poor clinical outcomes (72).

Interestingly, a new genome algorithm has been developed based on a prospectively sequenced cohort with 3,411 patients for application to panel targeted DNA sequencing data from AVC patients (73). It can be used to predict the outcomes of different AVC patients with mixed subtypes. Additionally, this algorithm was consistent with reports (45, 48), indicating that the genomic scores were higher in patients with the pancreaticobiliary subtype (mutations of the *KRAS* gene) and associated with lower survival probability.

### 4.8 Limitations of molecular classification

There are many advantages to the molecular classification of AVC. However, some complex limitations in gene testing and analysis for AVC should be noted. First, the incidence of AVC is low, and the heterogeneity of AVC patients, such as histological origins, is substantial. Therefore, the available samples for gene testing are relatively few, and reliable data in large sample size research are absent. Second, the different methods of gene testing and various definitions of gene mutations could make equivalent analysis very difficult in clinical practice. Third, the role of molecular alterations in the modifications of therapy may be limited. Only 13.3% (397/2,984) of patients harbored the pathogenic germline variant (PGV), and nearly 30% of those patients with high-penetrance variants of PGV had modifications in their therapy (74). In summary, the molecular classification data for AVC patients are hard to come by, and not every finding regarding molecular characteristics in AVC is accurate and has therapeutic applicability. However, the NCCN recommends testing some valuable genes. Therefore, focusing on those key molecular alterations in clinical practice is relatively cost-effective.

## 5 Future prospects

The close relationships between histological subtypes, molecular features, and clinical outcomes of AVC patients would be proven. It was mentioned that some other factors, such as safer surgical therapy (75) and efficient management in the perioperative period (76), could be included in the prognostic analysis for reaching reliable conclusions. With the rapid accumulation of data, the development of data sharing platforms, and the decrease of the cost of gene testing and analysis, the conclusions will be verified in the near future.

## 6 Conclusions

The histological subtypes and molecular alterations of patients with AVC play an important role in predicting and improving clinical outcomes. In the analysis of the histological subtypes of AVC, the pancreaticobiliary subtype is obviously related to poor clinical outcomes. Immunohistochemical examinations are the key to prognosis analysis. Testing the molecular characteristics of tumors not only helps to predict the prognosis of patients with mixed or unknown histological subtypes but also provides information for selecting targeted treatments for AVC patients.

## Author contributions

HL: conceptualization, methodology and investigation, formal analysis, and writing of the original draft. YZ: acquisition of data and article revision. Y-kW: conceptualization, methodology, supervision, and article revision. All authors approved the final manuscript and were accountable for the contents of the article. All authors contributed to the article and approved the submitted version.
